# Response of soil fungal communities and their co-occurrence patterns to grazing exclusion in different grassland types

**DOI:** 10.3389/fmicb.2024.1404633

**Published:** 2024-07-03

**Authors:** Anjing Jiang, Yiqiang Dong, Julihaiti Asitaiken, Shijie Zhou, Tingting Nie, Yue Wu, Zeyu Liu, Shazhou An, Kailun Yang

**Affiliations:** ^1^College of Grassland Science, Xinjiang Agricultural University, Ürümqi, China; ^2^Xinjiang Key Laboratory of Grassland Resources and Ecology, Ürümqi, Xinjiang, China; ^3^Key Laboratory of Grassland Resources and Ecology of Western Arid Region, Ministry of Education, Ürümqi, China; ^4^Postdoctoral Mobile Station of Xinjiang Agricultural University, Ürümqi, China; ^5^College of Animal Science, Xinjiang Agricultural University, Urumqi, Xinjiang, China

**Keywords:** grazing exclusion, soil fungi, Grassland type, fungal diversity, soil nutrients

## Abstract

Overgrazing and climate change are the main causes of grassland degradation, and grazing exclusion is one of the most common measures for restoring degraded grasslands worldwide. Soil fungi can respond rapidly to environmental stresses, but the response of different grassland types to grazing control has not been uniformly determined. Three grassland types (temperate desert, temperate steppe grassland, and mountain meadow) that were closed for grazing exclusion for 9 years were used to study the effects of grazing exclusion on soil nutrients as well as fungal community structure in the three grassland types. The results showed that (1) in the 0–5 cm soil layer, grazing exclusion significantly affected the soil water content of the three grassland types (*P* < 0.05), and the pH, total phosphorous (TP), and nitrogen-to-phosphorous ratio (N/P) changed significantly in all three grassland types (*P* < 0.05). Significant changes in soil nutrients in the 5–10 cm soil layer after grazing exclusion occurred in the mountain meadow grasslands (*P* < 0.05), but not in the temperate desert and temperate steppe grasslands. (2) For the different grassland types, Archaeorhizomycetes was most abundant in the montane meadows, and Dothideomycetes was most abundant in the temperate desert grasslands and was significantly more abundant than in the remaining two grassland types (*P* < 0.05). Grazing exclusion led to insignificant changes in the dominant soil fungal phyla and α diversity, but significant changes in the β diversity of soil fungi (*P* < 0.05). (3) Grazing exclusion areas have higher mean clustering coefficients and modularity classes than grazing areas. In particular, the highest modularity class is found in temperate steppe grassland grazing exclusion areas. (4) We also found that pH is the main driving factor affecting soil fungal community structure, that plant coverage is a key environmental factor affecting soil community composition, and that grazing exclusion indirectly affects soil fungal communities by affecting soil nutrients. The above results suggest that grazing exclusion may regulate microbial ecological processes by changing the soil fungal β diversity in the three grassland types. Grazing exclusion is not conducive to the recovery of soil nutrients in areas with mountain grassland but improves the stability of soil fungi in temperate steppe grassland. Therefore, the type of degraded grassland should be considered when formulating suitable restoration programmes when grazing exclusion measures are implemented. The results of this study provide new insights into the response of soil fungal communities to grazing exclusion, providing a theoretical basis for the management of degraded grassland restoration.

## 1 Introduction

Grazing is a major grassland utilization strategy that comes with certain economic effects and environmental consequences (Yin et al., 2021), such as grassland degradation due to interactions with changing climatic conditions, slowing vegetation growth (Dlamini et al., 2016), altering the soil structure, and significantly affecting ecosystem services such as grassland windbreaks and sand stabilization, water retention, and carbon sequestration functions (Zhao et al., 2020). Grassland degradation has become an important ecological problem worldwide and has received increasing attention from ecologists (Bardgett et al., 2021; Wang et al., 2022c). Grazing exclusion is an effective way to restore degraded grasslands by relieving grazing pressure and promoting the self-recovery of degraded grasslands (Liu et al., 2020; Sun et al., 2021). Most of the previous studies on the effects of grazing bans on soil microorganisms have focussed on soil bacteria (Wang et al., 2022a). However, fungi, which are directly dependent on plant communities, are more sensitive to changes in soil nutrients and have stronger aboveground and belowground interactions (Millard and Singh, 2010). Therefore, research on the effects of grazing exclusion on soil fungal communities is highly important.

Soil fungi, as the second most important group in the soil microbial community and decomposers in the ecosystem, play an important role in promoting the uptake of various nutrients by vegetation, improving soil structure, participating in the degradation of apoplastic matter, promoting the turnover of nutrients during cycling and other ecological processes (Tedersoo et al., 2014; Peay et al., 2016). In addition, soil fungal communities are affected by different environmental factors, can adapt dynamically to the environment, and their composition can reflect the ecological status of the soil (Li et al., 2011). Therefore, studying the effects of grazing bans on soil fungal communities and understanding the drivers that influence soil fungal communities are critical for improving our understanding of ecosystem restoration mechanisms. However, the restoration of degraded grasslands by grazing exclusion is controversial. For example, Zhang et al. (2018) reported that grazing exclusion leads to a decrease in fungal diversity in a study on semiarid grasslands. Studies on meadow grasslands (Kaurin et al., 2018) and temperate steppe grasslands (Ma et al., 2023) reported that grazing exclusion favored an increase in fungal diversity, whereas a study on *Seriphidium transiliense* desert grasslands (Li et al., 2023) reported that the response of soil MBC, MBN, and MBP to grazing exclusion was not significant. These results suggest that no consensus exists on the effect of grazing exclusion on grassland soil fungal communities, which may be because most of the previous studies focussed on one grassland type and few studies considered different habitat conditions and their effect on soil microbial responses. Whether these differences are caused by different biotic and abiotic factors in the context of a wider variety of biotic communities remains to be investigated.

The Tian Shan Mountain Range is one of the seven major mountain systems in the world, with complex topography and remarkable geomorphological features, leading to obvious differences in climate, soil conditions, and vegetation cover in mountainous areas, with multiple types of desert–alpine meadows, which play a more important role in grassland ecosystems (Li et al., 2022) and is an ideal area for the study of different grassland types. Numerous studies (Asitaiken et al., 2021; Zhou et al., 2022) have shown that grazing exclusion promotes the recovery of vegetation and soil nutrients in degraded grasslands in the Tian Shan Mountains, but there is a lack of research on the effects of grazing exclusion on soil fungal communities.

To address the above questions, this study selected three different grassland types (temperate desert grassland, temperate steppe grassland, and mountain meadow grassland) in the Tian Shan Mountains as the research object, studied the effects of grazing exclusion on soil nutrients and soil fungal communities in different grassland types, and proposed two scientific questions: (1) does grazing exclusion have a differential effect on soil fungal communities in the three grassland types? and (2) what drives changes in soil fungal diversity, and how does grazing exclusion affect soil fungal communities through changes in plant communities and soil nutrients?

## 2 Materials and methods

### 2.1 Study area

**Figure 1:**
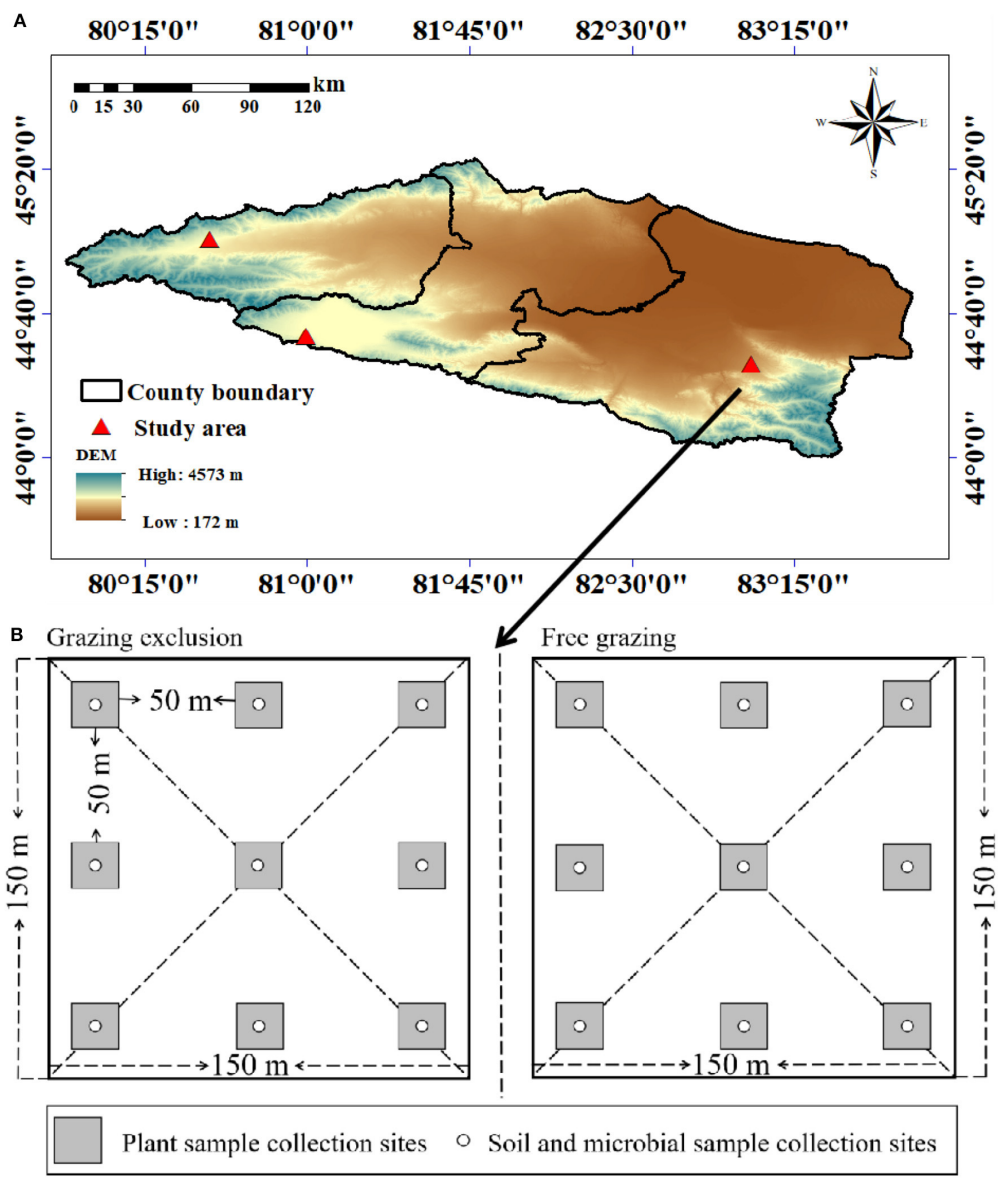
Digital elevation map showing the locations of the three sampled sites in the Tianshan Mountains **(A)**. **(B)** shows the sample layout.

The study areas are located in Bortala Mongol Autonomous Prefecture, Xinjiang (44°02′-45°23′N, 79°53′-83°53′E, altitude 189–4,569 m), state-level fixed monitoring sites in Bole city (mountain meadow grassland), Wenquan city (temperate steppe grassland), and Jinghe city (temperate desert grassland) (Figure 1A). With an average annual temperature of 1.1–7.8°C and average annual precipitation of 102–400 mm, the dominant species in these areas are *Alchemilla tianschanica, Stipa capillata*, and *Seriphidium borotalense* (Table 1).

**Table 1:** Description of the study sites.

**Sites**	**Grassland types**	**Dominant species**	**geographical position**	**Elevation (m)**	**Enclosure year**
Jinghe County	Temperate desert	*Seriphidium borotalense+Suaeda glauca*	E83.05°, N44.43°	1,178	2012
Wenquan County	Temperate steppe	*Stipa capillata+Artemisia frigida*	E80.55°, N45.01°	2,338	2012
Bole Country	Mountain meadow	*Alchemilla tianschanica+Elytrigia repens+Bromus inermis*	E80.99°, N44.56°	1,017	2012

### 2.2 Experimental design and field sampling

Three grassland types were selected in July 2021: temperate desert, temperate steppe, and mountain meadow. Long-term free-grazing sample plots and grazing-excluded plots were selected for each research area. Three sample strips were laid out in each sample plot, and the spacing between the sample zones was >50 m. In each sampling zone, three 1 m × 1 m sample plots were set up, with a spacing of 50 m, and the total number of sample plots was 54 (Figure 1B). The plants in each sample plot were recorded, as were the plant height, cover, density, and biomass. Soil samples were taken using the soil auger method. In the sample plot where the characteristics of the grassland community were measured, soil samples were taken using a soil auger at soil depths of 0–5 and 5–10 cm in layers, and each sample line was evenly mixed, put into a labeled Ziplock bag, and returned to the laboratory. Some of the samples were kept in the refrigerator at 4°C, whilst the remaining samples were naturally dried indoors after plant roots, gravel, and other debris were removed. The soil samples were then ground, mixed, and sieved through a 1 or 0.25 mm sieve for storage for laboratory analysis.

### 2.3 Sample analysis

#### 2.3.1 Plant analyses

The natural height (cm) of five random plants of each species, or as many as were present if there were fewer than five plants, and the mean value was calculated. Species cover (%) was determined using the projection method. Density was determined using the direct counting method by recording the number of occurrences of the same plant or clumps of the same plant within the sample plots of each species (plant/m^2^). Aboveground biomass was determined using the flush mowing method. The fresh weights were recorded, placed into envelopes, and brought back to the laboratory to kill the greening at 105°C for 30 min. The samples were then baked at 80°C until the weight became constant, and the biomass was weighed (g/m^2^).

The α-diversity indices selected for this study were the Shannon–Wiener, Patrick's, Simpson's, and Pielou's, and the following equations were used to estimate the diversity of the plant communities (Wu et al., 2009) (Supplementary Figure S1).


(1) $$\begin{array}{l} \text{Importance value }\left(\text{IV}\right):IV_{i}\;=\;\left(H_{ri}\;+\;C_{ri}+\;D_{ri}+\;B_{ri}\right)/4 \end{array}$$

(2) $$\begin{array}{l} \text{Patrick richness index }\left(\text{R}\right):R\;=\;S \end{array}$$

(3) $$\begin{array}{l} \text{Simpson dominance index }\left(\text{D}\right):D\;=\;1\;-\;\sum {IV}_{i}^{2} \end{array}$$

(4) $$\begin{array}{l} \text{Shannon–Wiener diversity index }\left(\text{H}\right):H=-\sum IV_{i}\ln{IV}_{i} \end{array}$$

(5) $$\begin{array}{l} \text{Pielou evenness index }\left(\text{E}\right):E=\left(-\sum IV_{i}\ln{IV}_{i}\right)/lnS \end{array}$$

where *H*_*ri*_ represents the relative height, *C*_*ri*_ indicates the relative coverage, *D*_*ri*_ represents the relative density, *B*_*ri*_ represents the relative biomass, S represents the total number of species in the plot, and *IV*_i_ represents the importance of the i species.

#### 2.3.2 Soil nutrient analyses

Soil pH and conductivity were recorded (water–soil ratio of 5:1) (Chen et al., 2022), and soil moisture content was determined by drying and weighing at 105°C for 24 h (Wang et al., 2023). Soil bulk density was measured gravimetrically after oven-drying (105°C, 24 h). Soil organic carbon, total nitrogen (TN), and total phosphorus (TP) contents were determined sequentially using the dichromate oxidation method, Kjeldahl method, and Mo–Sb colorimetric method (Parkinson and Allen, 1975; Blakemore et al., 1987; Zhang et al., 2018).

#### 2.3.3 Determination and analysis of soil fungal communities

DNA extraction and PCR amplification: total genomic DNA from the samples was extracted using the CTAB method. The DNA concentration and purity were monitored on 1% agarose gels. According to the concentration, DNA was diluted to 1 ng/μL using sterile water. Internal transcribed spacer (ITS) rRNA genes of distinct regions were amplified using specific primers (ITS1-1F-F (5′-CTTGGTCATTTAGAGGAAGTAA-3′) and ITS1-1F-R (5′-GCTGCGTTCTTCATCGATGC-3′) with a barcode (Ghannoum et al., 2010). All PCRs were carried out using 15 μL of Phusion^®^ High-Fidelity PCR Master Mix (New England Biolabs), 2 μM forward and reverse primers, and approximately 10 ng of template DNA. Thermal cycling consisted of initial denaturation at 98°C for 1 min, followed by 30 cycles of denaturation at 98°C for 10 s, annealing at 50°C for 30 s, and elongation at 72°C for 30 s. Finally, the samples were incubated at 72°C for 5 min. The same 1X TAE buffer was mixed with the PCR products, and electrophoresis was performed on a 2% agarose gel for detection. The PCR products were mixed in equal ratios and purified using a Qiagen Gel Extraction Kit (Qiagen, Germany).

Illumina NovaSeq sequencing: Sequencing libraries were generated using a TruSeq^®^ DNA PCR-Free Sample Preparation Kit (Illumina, USA) following the manufacturer's recommendations, and index codes were added. The library quality was assessed on a Qubit@ 2.0 fluorometer (Thermo Scientific). Finally, the library was sequenced on an Illumina NovaSeq platform, and 250 bp paired-end reads were generated.

### 2.4 Statistical analysis

All the data are expressed as means and standard errors. The data were preprocessed using Excel 2019, and independent sample *t*-tests and one-way ANOVA for plant community characteristics, soil nutrients, the abundance of dominant soil fungi, and diversity were performed using SPSS 25 software. Origin 2021 (OriginLab Corporation, USA) was subsequently used for histogram plotting. The beta diversity of fungal communities was estimated using Bray–Curtis distances in the “vegan” package (Oksanen et al., 2020) and plotted using the ggplot2 package in R 4.2.1 (Wilkinson, 2011), and permutational multivariate analysis of variance was used to test whether grazing exclusion, different grassland types, and their interactions had significant effects on the soil fungal community composition. In addition, to demonstrate more intuitively the effects of plant communities and soil nutrients on soil fungal communities, we selected soil fungal diversity indicators to perform a Mantel test with each of the soil nutrient indicators, and Mantel correlation was used to assess the relationships between soil fungal diversity and soil environmental variables. To further elucidate the effects of plant communities and soil nutrients on the main class of fungi, redundancy analyses were performed using R software after data normalization and variable commonality tests. Both the Mantel test and RDA were performed in the “vegan” package in R 4.2.1 (Yin et al., 2021; Wang et al., 2023). For the soil fungal symbiotic network, first, operational taxonomic units (OTUs) with a relative abundance of < 0.1% were removed to reduce the number of rare OTUs. Second, the correlation coefficients between OTUs were calculated using Pearson's *R* > 0.7, and *P* < 0.05 as the limiting factors, and the correlation coefficient between OTUs was calculated using the corr.test() function in the “psych” package (Jiao et al., 2022). Finally, topological parameters, including the average degree, average path length, clustering coefficient, and modularity were extracted for each treatment to assess the response of the soil fungal symbiotic network patterns to grazing bans as a function of different grassland types, and the network was visualized using Gephi 0.10. Structural equation models (SEMs) were constructed using the lavaan package (Rosseel, 2011), and the models included grazing exclusion, plant community characteristics, soil nutrients, and fungal community characteristics.

## 3 Results

### 3.1 Soil nutrients

Grazing exclusion significantly reduced the soil moisture content in the 0–5 cm soil layer of the three grassland types compared to the grazing area (*P* < 0.05) (Table 2). For the mountain meadow grassland, grazing significantly reduced the soil organic carbon, TP, carbon-to-nitrogen ratio, and carbon-to-phosphorus ratio and significantly increased the soil pH, electrical conductivity, and bulk density (*P* < 0.05). However, the soil electrical conductivity increased significantly (*P* < 0.05) in the 0–5 cm soil layer of the temperate steppe grassland. For the 5–10 cm soil layer, the physicochemical properties of the soil changed significantly (*P* < 0.05) after grazing exclusion in the mountain meadow grassland, whilst the changes in the other grassland types were not significant. The soil moisture content, organic carbon, and TN in the 0–10 cm soil layer of mountain meadow grassland were significantly greater than those of temperate desert and temperate steppe grasslands, and the pH of the 0–10 cm soil layer changed significantly amongst the three grassland types. Overall, grazing exclusion had a significant effect on the soil physicochemical properties in the mountain meadow grassland.

**Table 2:** Differences in soil chemical properties between the grazing and grazing exclusion treatments.

**Soil layer(cm)**	**Grassland type**	**Enclosure treatment**	**pH**	**EC(ms•m^−1^)**	**BD(g•cm^−3^)**	**SM(%)**	**SOC(g•kg^−1^)**	**TN(g•kg^−1^)**	**TP(g•kg^−1^)**	**C/N**	**C/P**	**N/P**
0~5	TD	GE	8.83 ± 0.12Aa	159.03 ± 53.73Aa	1.38 ± 0.07Aa	**2.08** **±0.11Aa**	5.92 ± 1.86Aa	0.67 ± 0.10Aa	0.82 ± 0.02Aa	9.84 ± 3.73Aa	7.33 ± 2.38Aa	0.82 ± 0.12Aa
		FG	8.80 ± 0.43Aa	285.00 ± 21.50Aa	1.36 ± 0.04Aa	**2.90** **±0.14Ab**	6.18 ± 0.57Aa	0.77 ± 0.07Aa	0.80 ± 0.01Aa	7.98 ± 0.07Aa	7.77 ± 0.81Aa	0.97 ± 0.10Aa
	TS	GE	7.64 ± 0.08Ba	**121.65** **±12.41Ba**	1.26 ± 0.08Aa	**3.01** **±0.37Aa**	7.41 ± 3.86Aa	2.38 ± 0.12Aa	0.48 ± 0.02Ba	2.98 ± 1.46Aa	14.93 ± 7.65Aa	4.91 ± 0.12Ba
		FG	7.67 ± 0.16Ba	**81.30** **±7.27Bb**	1.31 ± 0.08Aa	**4.63** **±0.31Ab**	14.73 ± 0.35Aa	2.31 ± 0.12Aa	0.51 ± 0.03Ba	6.39 ± 0.22Aa	29.21 ± 2.35Aa	4.61 ± 0.50Ba
	MM	GE	**7.41** **±0.07Ca**	**185.97** **±1.90ABa**	**0.87** **±0.04Ba**	**19.00** **±0.52Ba**	**29.29** **±2.12Ba**	8.33 ± 1.47Ba	**0.93** **±0.04Ca**	**3.69** **±0.58Aa**	**31.77** **±3.31Ba**	8.97 ± 1.58Ca
		FG	**6.56** **±0.09Cb**	**136.12** **±5.94ABb**	**0.68** **±0.01Bb**	**34.96** **±2.48Bb**	**107.56** **±14.40Bb**	10.30 ± 1.33Ba	**1.23** **±0.09Cb**	**10.43** **±0.21Ab**	**87.14** **±8.66Bb**	8.34 ± 0.70Ca
5~10	TD	GE	9.41 ± 0.09Aa	195.25 ± 48.63Aa	1.32 ± 0.06Aa	3.71 ± 0.87Aa	5.33 ± 2.23Aa	0.51 ± 0.05Aa	0.75 ± 0.05Aa	10.84 ± 4.22Aa	7.24 ± 2.93Aa	0.67 ± 0.03Aa
		FG	9.45 ± 0.12Aa	267.83 ± 19.39Aa	1.34 ± 0.07Aa	1.67 ± 0.33Aa	4.20 ± 0.54Aa	0.59 ± 0.08Aa	0.85 ± 0.01Aa	7.34 ± 0.97Aa	4.97 ± 0.67Aa	0.69 ± 0.10Aa
	TS	GE	7.30 ± 0.21Ba	149.73 ± 4.00Ba	1.15 ± 0.08ABa	6.06 ± 0.81Aa	12.93 ± 5.97Aa	3.17 ± 0.17Ba	0.54 ± 0.03Ba	4.01 ± 1.87Aa	24.47 ± 11.67Ba	5.90 ± 0.24Ba
		FG	7.64 ± 0.04Ba	126.32 ± 9.11Ba	1.27 ± 0.09ABa	5.83 ± 0.98Aa	16.15 ± 0.97Aa	2.74 ± 0.28Ba	0.53 ± 0.01Ba	6.05 ± 0.81Aa	30.79 ± 2.16Ba	5.22 ± 0.54Ba
	MM	GE	**7.42** **±0.06Ca**	**140.82** **±4.16Ba**	**1.17** **±0.05Ba**	**12.52** **±1.64Ba**	**15.85** **±2.50Ba**	**3.51** **±0.26Ca**	**0.78** **±0.01Aa**	**4.54** **±0.70Aa**	**20.27** **±2.97Ba**	**4.49** **±0.26Ba**
		FG	**6.35** **±0.05Cb**	**89.55** **±6.19Bb**	**0.90** **±0.06Bb**	**23.67** **±1.90Bb**	**66.18** **±3.89Bb**	**7.70** **±0.45Cb**	**0.99** **±0.06Ab**	**8.60** **±0.00Ab**	**67.03** **±0.68Bb**	**7.79** **±0.08Bb**

Different capital letters indicate that soil nutrients varied significantly amongst different grassland types, lowercase letters indicate that different treatments had a significant effect on soil nutrients in the same grassland type, and bolded letters indicate that they varied significantly amongst different treatments (P < 0.05). TD, temperate desert grassland; TS, temperate steppe grassland; MM, mountain meadow grassland, GE, grazing exclusion; FG, freely grazing; EC, electric conductivity; BD, bulk density; SM, soil moisture; SOC, soil organic carbon; TN, total nitrogen; TP, total phosphorus; C:N, carbon:nitrogen; C:P, carbon:phosphorus; N:P, nitrogen:phosphorus.

### 3.2 Soil fungal community diversity and composition

**Figure 2:**
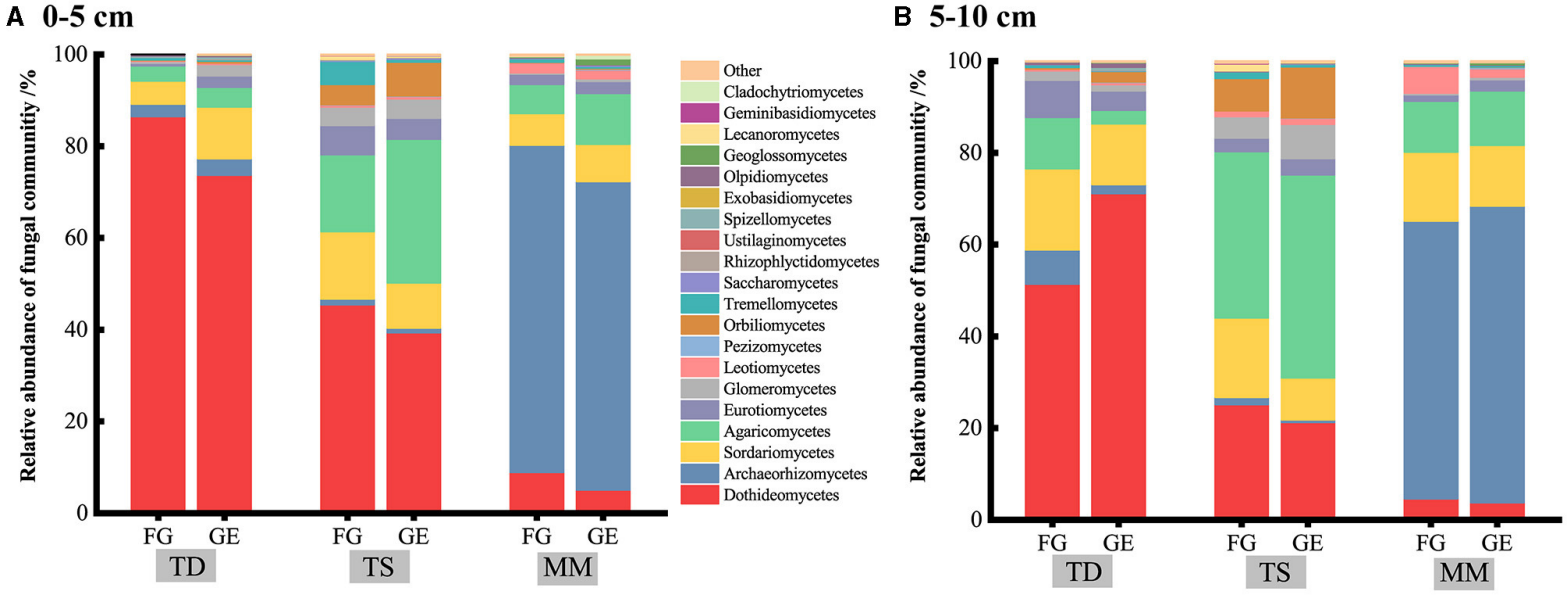
Stacked bar chart shows the relative abundance of fungi at the class level. **(A, B)** represent the effects of grazing exclusion on the soil fungal classes of different grassland types in the 0–5 cm and 5–10 cm soil layers, respectively.

**Figure 3:**
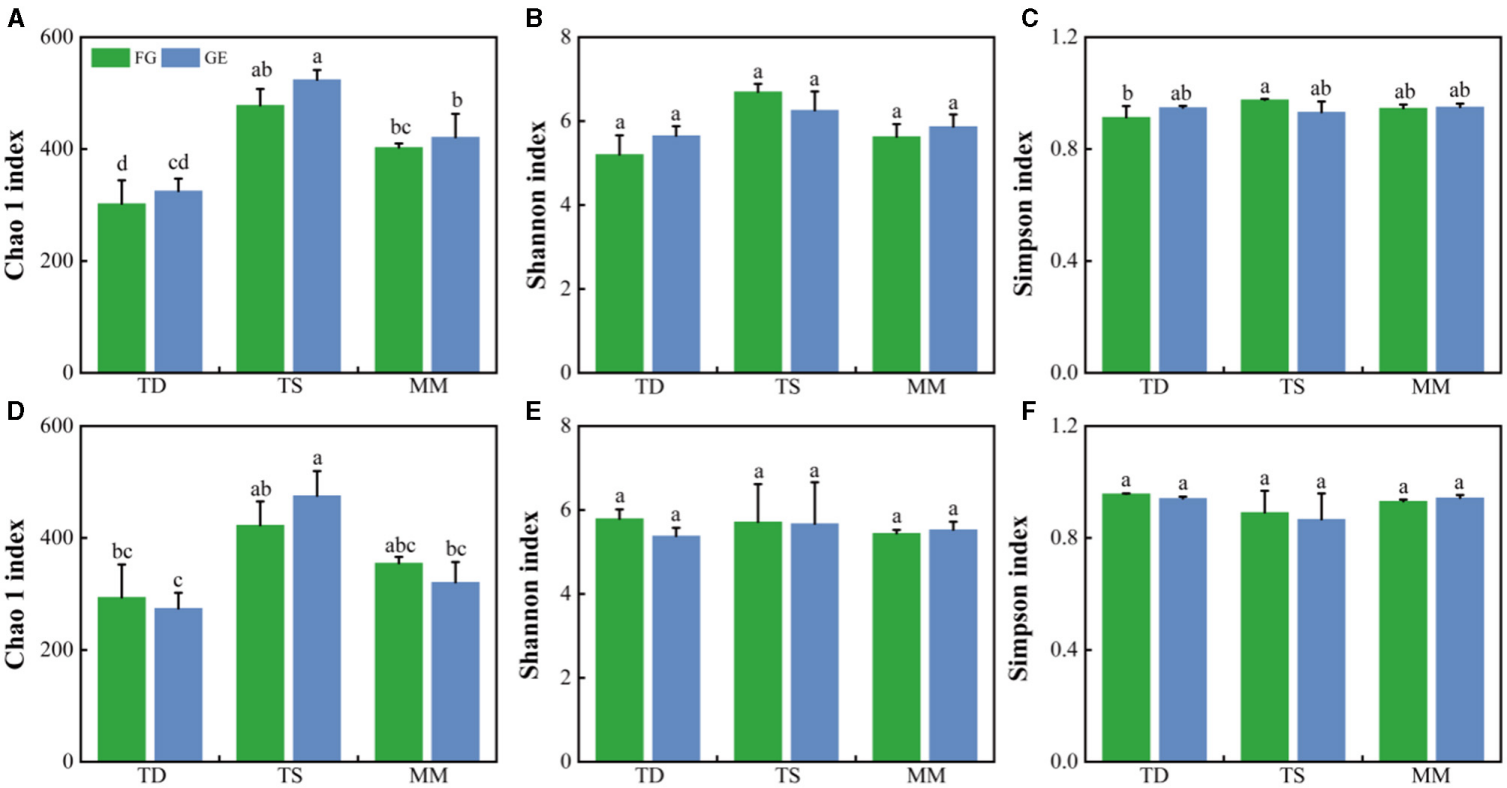
Fungal α diversity **(A–C)** of the 0–5 cm soil layer based on the Chao 1 index, Shannon index, and Simpson index; **(D–F)** of the 5–10 cm soil layer based on the Chao 1 index, Shannon index, and Simpson index. The error bars indicate standard errors (three replicate sites). Different lowercase letters indicate significant differences (*P* < 0.05) between grazing and exclusion conditions for different grassland types. The same lowercase letter indicates that there is no significant difference between grazing and exclusion in different grassland types.

At the phylum level, soil Dothideomycetes, Archaeorhizomycetes, Agaricomycetes, and Sordariomycetes were the dominant fungal groups (Figure 2). The relative abundance of Archaeorhizomycetes in the 0–10 cm soil layer of both the grazing and grazing exclusion samples in the mountain meadow grassland was significantly greater than that in the temperate desert and temperate steppe grasslands (*P* < 0.05), whereas the relative abundance of Dothideomycetes in the temperate desert grassland was significantly greater than that in the other two grasslands (*P* < 0.05) (Supplementary Table S1). The abundance of other dominant fungi did not differ significantly amongst the three grassland types, and the changes in the relative abundance of dominant fungi were not significant after grazing exclusion compared to those in the grazing areas. The effects of grazing exclusion on the fungal diversity indices of the three grassland types were also not significant (Figures 3A–F). The Chao 1 index of the temperate steppe grassland was significantly greater than that of the other grasslands after grazing exclusion only (*P* < 0.05).

**Figure 4:**
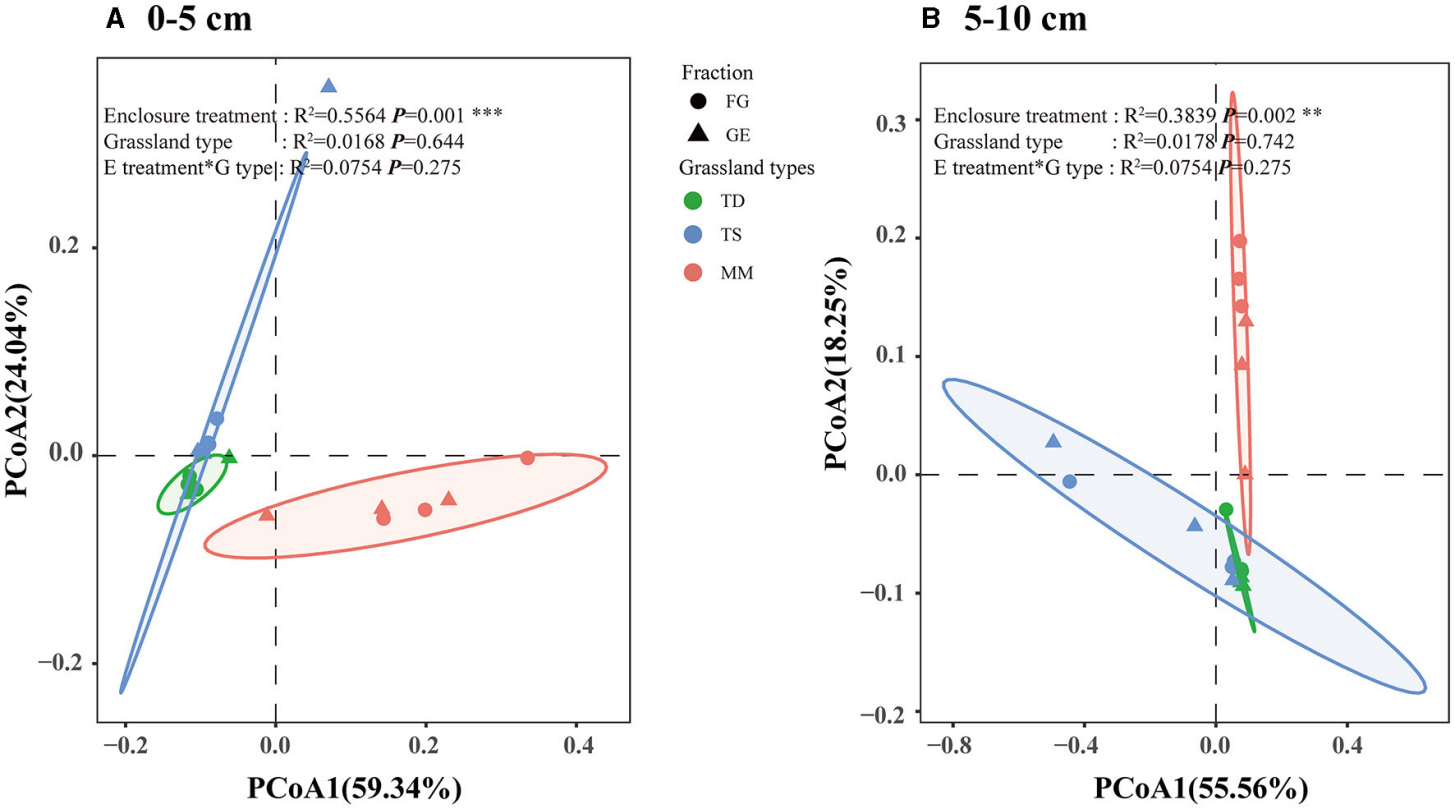
Fungal community structure assessed by β diversity patterns using principal coordinate analysis plots of Bray–Curtis distances. The different colors represent exclusion or grazing soils, and the shapes represent grassland types: temperate desert, temperate steppe, and meadow steppe. ANOSIM was used to test the significance between groups. **(A)** of the 0–5 cm soil layer based on the β diversity, **(B)** of the 5–10 cm soil layer based on the β diversity.

Based on ANOSIM analyses, soil fungal samples from Bray–Curtis distances in the grazing exclusion and grazing areas were significantly separated from each other in both the 0–5 cm soil layer and the 5–10 cm soil layer, indicating that soil fungal β diversity changed significantly after grazing exclusion (R^2^ = 0.5564, *P* = 0.001, and R^2^ = 0.3839, *P* = 0.002, respectively) (Figures 4A, B). The changes in soil fungal β diversity amongst different grassland types were not significant, and the interaction between grazing exclusion and different grassland types did not significantly affect the soil fungal community. Overall, grazing exclusion did not significantly affect the relative abundance or α diversity of fungal classes but did significantly affect the β diversity of fungi.

### 3.3 Soil fungal co-occurrence patterns

**Figure 5:**
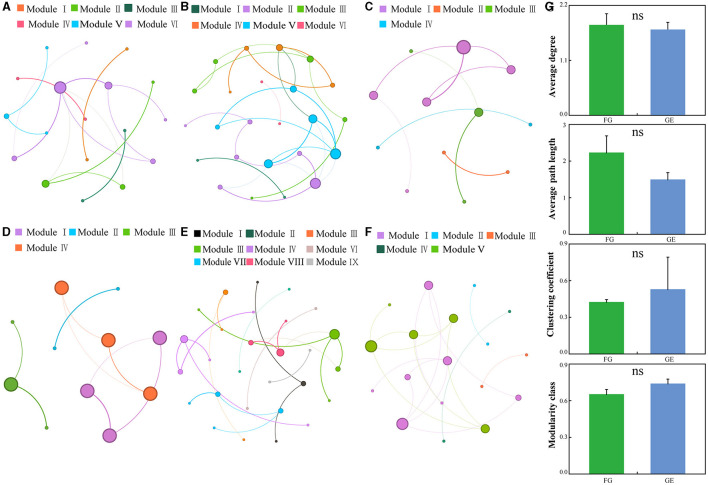
Overall co-occurrence networks of soil fungi and changes in topological parameters of soil fungi across different treatments and growth stages. The node size indicates the connectivity degree. The colors of the nodes and edges are grouped by modularity class. Different colors refer to different modules. ns indicates a *P* > 0.05. **(A–C)** indicate grazing, **(D–F)** indicate grazing exclusion. **(G)** is the network topological indexes.

In this study, co-occurrence networks were constructed for soil fungal communities in grazing and grazing exclusion treatments. Compared to those in the grazing exclusion area, the soil fungal communities in the grazing area had greater average degrees and average path lengths. The grazing exclusion area had greater clustering coefficients and modularity classes than did the grazing area (Figures 5A–G). All three grassland types exhibited a highly modular structure (modularity > 0.59) under both the grazing and grazing exclusion treatments. In addition, the co-occurring network of temperate steppe grassland under the grazing exclusion treatment exhibited nine network modules, whilst the other grassland types exhibited only four to six network modules.

### 3.4 Relationships between soil nutrients and the fungal community

**Figure 6:**
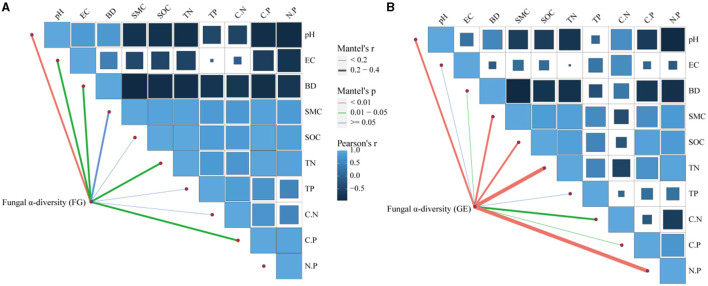
Mantel correlations between the community compositions of soil fungi and environmental factors. **(A)** of the 0–5 cm soil layer, **(B)** of the 5–10 cm soil layer.

**Figure 7:**
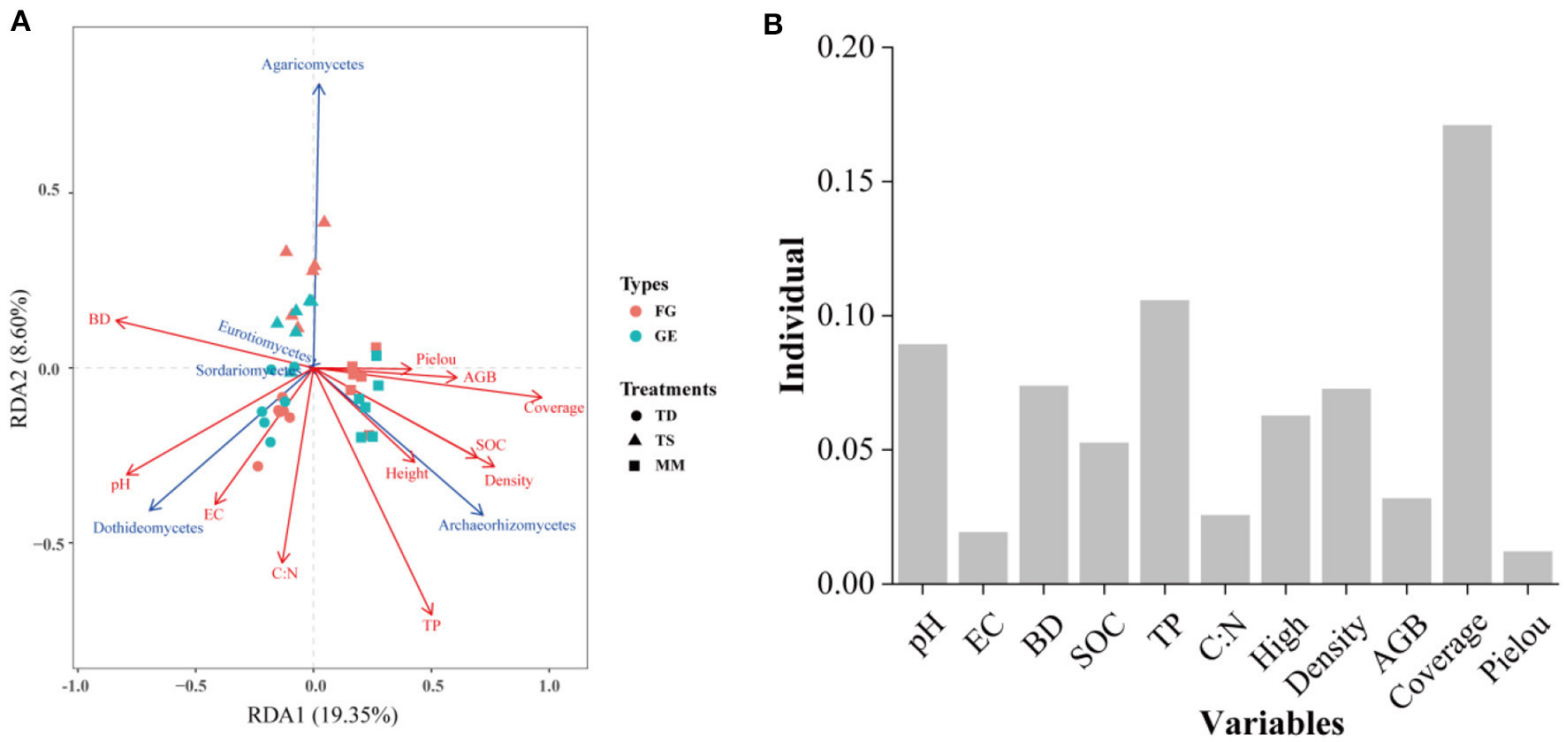
Redundancy analysis of the effect of significant soil nutrients on the composition of the fungal community at the class level **(A)**. Red arrows refer to soil properties, and blue solid lines refer to fungal classes. Environmental factors and their interpretation rates of the variations in fungal community components **(B)**.

The correlations between soil fungal diversity and nutrients are shown in Figure 6, which shows that the drivers of diversity were soil N:P, TN, BD, SMC, and C:P. Soil pH was an important environmental factor affecting the diversity of soil fungi in both grazing treatments. In addition, RDA was also used to further investigate the effects of vegetation community characteristics and soil physicochemical properties on the soil fungal community composition in the 0–10 cm soil layer (Figure 7). The percentages of the first and second axes in the 0–10 cm soil layer were 19.35% and 8.60%, respectively, and the cumulative interpretation rate reached 27.95%. Amongst these factors, Plant coverage had the greatest effect on the distribution of soil fungal communities, followed by TP, pH and BD.

### 3.5 Relationships between soil properties and the fungal community

**Figure 8:**
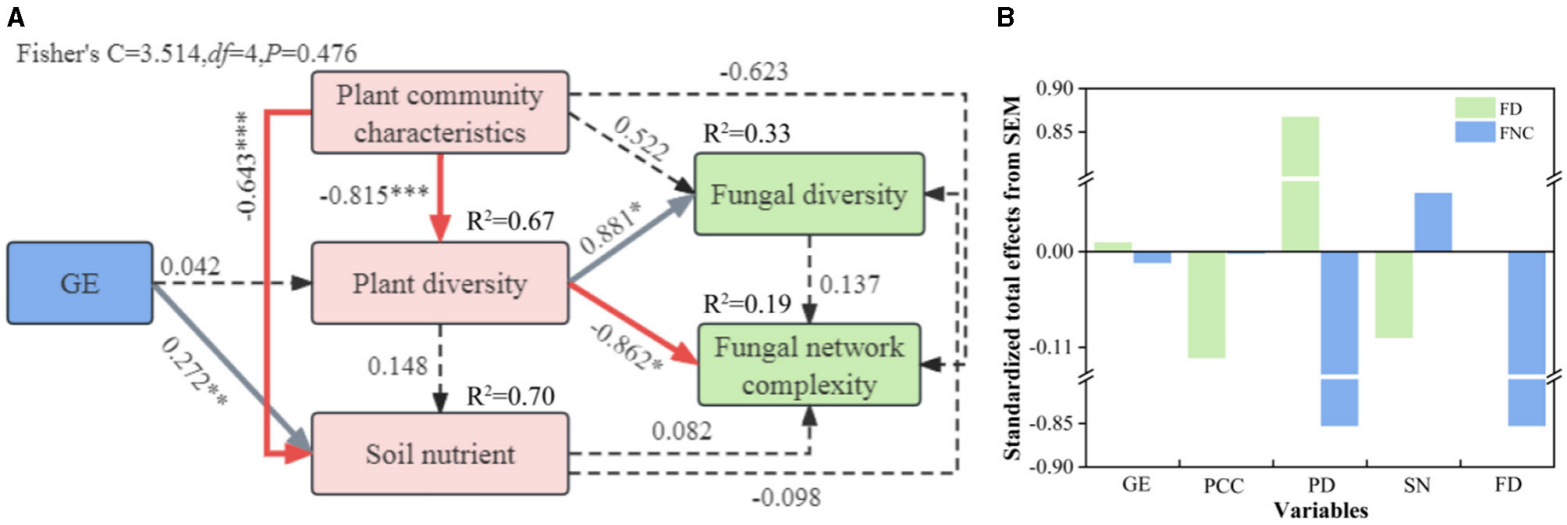
Structural equation modeling shows the direct and indirect effects of plant community characteristics, plant diversity, and soil nutrients on fungal diversity and network complexity **(A)**. Total effects of fraction, plant community characteristics, plant diversity, and soil nutrients on fungal diversity and network complexity **(B)**. The solid line indicates that the correlation is significant, and the dashed line indicates that the correlation is not significant. The gray and red arrows indicate positive and negative relationships, respectively. The numbers adjacent to the arrows represent the standardized path coefficients. FD, fungal diversity; FNC, fungal network complexity; PCC, plant community characteristics; PD, plant diversity; SN, soil nutrients.

Plant community characteristics and soil nutrients were used as predictors to establish SEMs by combining Mantel correlations and RDA to explore the effects of plant communities and soil nutrients on soil fungal α diversity as well as on symbiotic networks (Figure 8). The SEM results showed that grazing exclusion had a significant positive effect on soil nutrients in the grasslands, and plant composition affected the soil fungal communities by negatively influencing plant diversity (Figure 8A). Plant diversity had both significant positive and negative effects on fungal diversity and network complexity, respectively, and had the greatest total path effects, 0.881 and −0.852, respectively (Figure 8B).

## 4 Discussion

### 4.1 Changes in soil nutrients under grazing exclusion

In recent years, grassland degradation has increased, and grassland ecosystems have been severely damaged as a result of long-term overgrazing. In this study, the values of SOC and TP in the grazing exclusion areas of temperate desert, temperate steppe, and mountain meadow grassland were much lower than the mean values of soil SOC (29.51 g/kg) and TN (2.3 g/kg) in China (Zhang et al., 2022), which indicated that the nutrient contents of temperate desert grassland soils were lower and that the soils were poorer. A comparison of the mean values of soil TP (0.52–0.78 g/kg) in China revealed that, in comparison to temperate desert grassland soils, temperate steppe grassland soils had greater TN and lower TP. Some findings have shown that grazing exclusion promotes soil nutrient accumulation (Cheng et al., 2016) by reducing livestock foraging and trampling, which allows vegetation communities to grow and reproduce. This, in turn, increases carbon inputs due to an increase in aboveground biomass and litterfall (Du and Gao, 2021). It has also been shown that soil carbon loss is accelerated through soil microbial respiration due to the inputs of livestock feces and urine from grazing sample plots (Pang et al., 2018). Whilst Yuan et al. (2020) reported that grazing exclusion did not significantly affect organic carbon, the present study revealed reduced soil organic carbon in three grassland types, particularly for mountain meadow grasslands (*P* < 0.05), possibly because the improvement in soil moisture increased the input of soil carbon (Hu et al., 2016). But we found a significant reduction in the soil moisture content of the three grassland types, which led to a decrease in soil carbon input. When the depletion amount was greater than the accumulation amount, it resulted in an overall decrease in soil organic carbon (Li et al., 2023). Soil is an important carrier for vegetation growth, and the distribution and content of elements such as nitrogen and phosphorus can directly affect its nutrient status. In this study, the TN and TP contents of the soil in the grazing exclusion area of the mountain meadow grassland were significantly lower than those in the grazing area, consistent with the findings of Zhang et al. (2019) but not with those of Du and Gao (2021). This may vary depending on the geographical location of the forbidden grassland, the degree of degradation of the grassland, the number of years it has been forbidden to graze, and the climatic conditions. Changes in C, N, and P contents during nutrient cycling are considered important factors for ecological stability (Li et al., 2018), and it was observed in this study that soil C/N decreased after grazing exclusion and that low C/N ratios accelerated microbial decomposition of organic matter and increased the rate of nitrogen mineralisation (Springob and Kirchmann, 2003). This suggests that the treatment increased microbial diversity, which in turn increased the rate of decomposition.

Soil bulk density is an indicator of aeration that is positively correlated with density and is mainly affected by soil structure, grazing and trampling, and soil organic matter content. After grazing exclusion treatment completely eliminated the direct trampling by livestock and allowed the vegetation to recover, reduced compactness and increased pore space led to reduced soil bulk density of temperate desert and temperate steppe grasslands (Wang S. et al., 2018). Unlike the results of most studies in which grazing exclusion decreased soil pH (Yao et al., 2018; Ma et al., 2023), our results revealed that the treatment significantly increased soil pH in montane meadow grasslands compared to grazing areas, which is consistent with the findings of Zhang et al. (2017) on the response of soil pH to grazing exclusion in desertified grasslands. The reason may be because livestock in grazing areas excrete feces and urine as they forage, and the increased volume of livestock urine leads to an increase in the rate of cycling of soil ions, which increases the concentration of hydrogen ions in the top layer of the soil, resulting in a higher soil pH in grazing exclusion areas than in grazing areas (Woodbridge et al., 2014). Taken together, for mountain meadow grasslands, which are richer and more diverse in plant species, grazing exclusion is detrimental to the restoration of their grassland soils, but for temperate desert and temperate steppe grasslands, grazing exclusion improves the physical structure of grassland soils.

### 4.2 Effects of grazing exclusion on fungal communities and co-occurrence patterns

Soil fungi act as decomposers in ecosystems, effectively breaking down organic matter and humus and participating in the C and N cycles (Lv et al., 2023). In this study, Dothideomycetes, Archaeorhizomycetes, and Sordariomycetes, of the Ascomycota, and Agaricomycetes of the Agaricomycetes, were the dominant fungal groups of the three grassland types. Although the fungal response to grazing exclusion differed, the dominant groups were more or less the same. Ascomycota and Basidiomycetes have been shown to be dominant groups of soil fungi (Wang et al., 2022d), and studies on alpine meadows on the Tibetan Plateau have shown that the dominant community of soil fungi in degraded grasslands is Basidiomycetes (Li et al., 2021), which is consistent with the results of this paper. Additionally, Ascomycota is also found to be the dominant community of soil fungi in the globally sampled range (Tedersoo et al., 2014). Although both Ascomycota and Basidiomycetes have important roles in decomposing organic matter, their division of labor is different; Ascomycota usually decomposes decaying and complex organic matter in the soil, whilst Basidiomycetes mainly decomposes lignocellulose, which is difficult to degrade in apoplastic plant matter (Yao et al., 2017). In this study, grazing exclusion had no significant effect on the abundance or α diversity of the dominant soil fungi, possibly because the 9 years of treatment were short and the response of soil fungi to successional age was weak (Brown and Jumpponen, 2015); therefore, the effect of short-term grazing exclusion on the abundance and the α diversity of soil fungi was not significant. For example, Wang et al. (2019) studied 14 and 19 years of grazing exclusion in semiarid grasslands and reported that soil fungal diversity increased with increasing years of grazing restriction. β diversity analysis was used to compare differences in species composition between groups. The closer the distance between two groups on a coordinate plot, the more similar the composition of these two groups. Wang et al. (2023) reported that prolonged grazing exclusion altered the composition of fungal communities. According to the PCoA results, the soil fungal β diversity of all three grassland types was significantly altered under the grazing exclusion treatment, whereas the changes amongst the grassland types were not significant, consistent with the results of Chen et al. (2020). This suggests that grazing exclusion altered the composition of the soil fungal community and that the changes in the composition were closely related to microbial activities. Grazing exclusion could regulate microbial ecological processes by changing the fungal community composition rather than its abundance or diversity. This may be because plant nutrient uptake, amongst other factors, is strongly linked to soil fungi and plant communities. Grazing exclusion, on the other hand, affects the aboveground biomass (Lan et al., 2023) and changes the composition and structure of plant communities (Sigcha et al., 2018), amongst other factors. This leads to a change in plant nutrient requirements (Du and Gao, 2021), a change that is an important factor leading to changes in soil fungal community composition.

Microbial interactions can form a complex network that enables the effective transfer of energy, matter, and information between microorganisms that contribute to ecosystem function (Faust and Raes, 2012). Co-occurrence network analyses are used to assess how numerous species aggregate into different ecological clusters and to reveal the interactions between them (Berry and Widder, 2014). Studies have shown that positive connections indicate mutual synergistic relationships between microorganisms, whilst negative connections indicate competitive relationships between microorganisms (Wang X. et al., 2018; Blanchet et al., 2020). The connections in the soil fungal co-occurrence network in this study were all dominated by positive correlations, which is consistent with the findings of Duan et al. (2021). This result may indicate that co-operative relationships between soil fungal communities work together to resist external disturbances when the soil fungal community is subjected to environmental stress (Hernandez et al., 2021). For example, the ability of fungal hyphae to find nutrients is enhanced when there is a shortage of substrate (de Boer et al., 2005). Moreover, the soil fungal community in the grazing area had a greater average degree and average path length than that of the treated area, indicating that the community had greater connectivity. This study revealed that the average path length of the network was short, and the rate of information transfer between the species of the soil fungal network was fast (Zhou et al., 2011). These findings indicate that the fungal network response speed gradually accelerated when the environment changed from grazing treatment to grazing exclusion treatment, making the soil fungal community more susceptible to environmental changes. The temperate steppe grassland exhibited low connectivity and high modularity characteristics under the grazing exclusion treatment, indicating that the soil fungi in the temperate steppe grassland exhibited high stability under the grazing exclusion treatment. This is because the rich plant source resources and improved soil environment created more ecological niches for microorganisms after grazing exclusion (Chen et al., 2020; Lin et al., 2021). This was evident in the fact that all three grassland types with grazing exclusion had more modules than the grazing soils. A greater number of modules indicates a higher complexity of the soil fungal community, implying that the fungi had a stronger ability to resist external disturbances (Wang et al., 2022b). A greater diversity of modules, in turn, leads to a greater diversity of interactions (Wang et al., 2022a). Taken together, exclusion treatment increased the stability of temperate steppe grasslands, increased the rate of information transfer between the soil fungal networks of the three grassland types, and increased the diversity of interactions.

### 4.3 Factors influencing soil fungal community composition and co-occurrence networks

In most studies, changes in soil microbial diversity as well as co-occurrence networks are usually associated with environmental variables (Zhang et al., 2018; Jiao et al., 2022; Geng et al., 2023). Soil microorganisms are very sensitive to the environment in which they live and differences in grassland utilization, type of grazing livestock, vegetation composition, geography, climate, and soils can lead to changes in soil microorganisms (Yin et al., 2019), and differences in soil nutrients affect soil microbial habitats to varying degrees, leading to changes in microbial communities (Kaspari et al., 2017). Some studies have shown that soil fungal communities are closely related to soil nutrients (Wang et al., 2015). The SEM revealed that although grazing bans did not have a direct effect on fungal communities and their diversity, they could indirectly and positively affect them by altering soil nutrients and plant diversity. Plants affected fungal communities through their aboveground apoplastic matter, nutrients from their underground root system, and the carbon they provided (Cline et al., 2018; Zhang et al., 2019). Increased plant diversity resulted in increased formation of apoplastic material as well as underground root secretions, which led to increased soil fungal diversity (Thakur et al., 2015). Previous findings that the effect of soil on microbial diversity is more significant than that of plants (Shu et al., 2024) are not consistent with our results showing that plant diversity had the greatest total effect on soil fungal communities. The findings that there is a lag in the effect of plant communities on soil fungi (Zhang et al., 2018) are also at odds with our results, suggesting that 9 years of grazing exclusion may be sufficient for soil fungi to respond to plant diversity. Unlike fungal diversity, plant communities were significantly negatively correlated with soil fungal networks, whilst soil nutrients were positively correlated with fungal networks, consistent with previous studies (Chen et al., 2020). Soil fungal networks are affected by pH (Liu et al., 2023), phosphorus content (Li et al., 2020), and nitrogen content (Chen et al., 2020), and soil TN significantly affects soil fungal networks (Deng et al., 2020). Moreover, when the nitrogen content is low, soil fungal networks meet their needs through enhanced competition (Yuan et al., 2021). According to the RDA, soil TP was found to be the environmental factor influencing dominant soil fungal flora, possibly because soil microbial genetic structure requires more phosphorus, so the amount of phosphorus limits the abundance of dominant flora of the soil fungal community as well as the complexity of the network (Mori et al., 2018). In addition, soil phosphorus increases the effective soil nitrogen by facilitating nitrogen mineralisation, which in turn affects dominant soil fungi (Wang et al., 2022b). Pommier et al. (2018) found a strong correlation between TN content and the diversity of dominant taxa in fungal communities in a long-term nitrogen addition experiment on European grasslands, which is similar to the results of this paper's study that found soil TN to influence the diversity of fungal communities. In our study, soil bulk density was the main driver of soil fungal changes, and moist and permeable soils allowed for a richer environment for microorganisms and thus greater heterogeneity of living environments, a result similar to that of Jiao et al. (2019). This study also revealed that both the abundance of fungal dominant classes and fungal diversity were affected by soil pH, suggesting that it is a key limiting factor affecting the abundance, diversity, and network complexity of soil fungi under grazed exclusion conditions.

## 5 Conclusion

The results of this study showed that grazing exclusion did not cause significant changes in the soil fungal community α diversity (*P* > 0.05) but significantly altered the soil fungal β diversity (*P* < 0.05). In addition, temperate grassland soil fungi are more stability to grazing exclusion. Soil pH was found to be a key factor influencing the abundance, diversity, and network complexity of soil fungal communities in the three grassland types. We observed that grazing exclusion indirectly affected soil fungal communities by influencing soil nutrients and that plant diversity was significantly positively and negatively correlated with fungal diversity and network complexity, respectively. The results of this study provide a deeper understanding of the soil fungal community structure of different grassland types in response to grazing exclusion and a theoretical basis for the healthy restoration of degraded grasslands according to local conditions.

## Data availability statement

The original contributions presented in the study are publicly available. This data can be found here: https://doi.org/10.5061/dryad.bcc2fqzn0.

## Author contributions

AJ: Writing – original draft. YD: Writing – review & editing. JA: Writing – review & editing. SZ: Writing – review & editing. TN: Writing – review & editing. YW: Writing – review & editing. ZL: Writing – review & editing. SA: Writing – review & editing. KY: Writing – review & editing.
