# Supplementary material for: Response of soil fungal communities and their co-occurrence patterns to grazing exclusion in different grassland types
Source: Front Microbiol. 2024 Jul 3;15:1404633. doi: 10.3389/fmicb.2024.1404633 (PMC11256198; doi:10.3389/fmicb.2024.1404633)
Supplement: Supplementary file 1 [file Data_Sheet_1.pdf]

Supplementary Figure S1 Vegetation characteristics as affected by grazing exc lusion and grassland type.

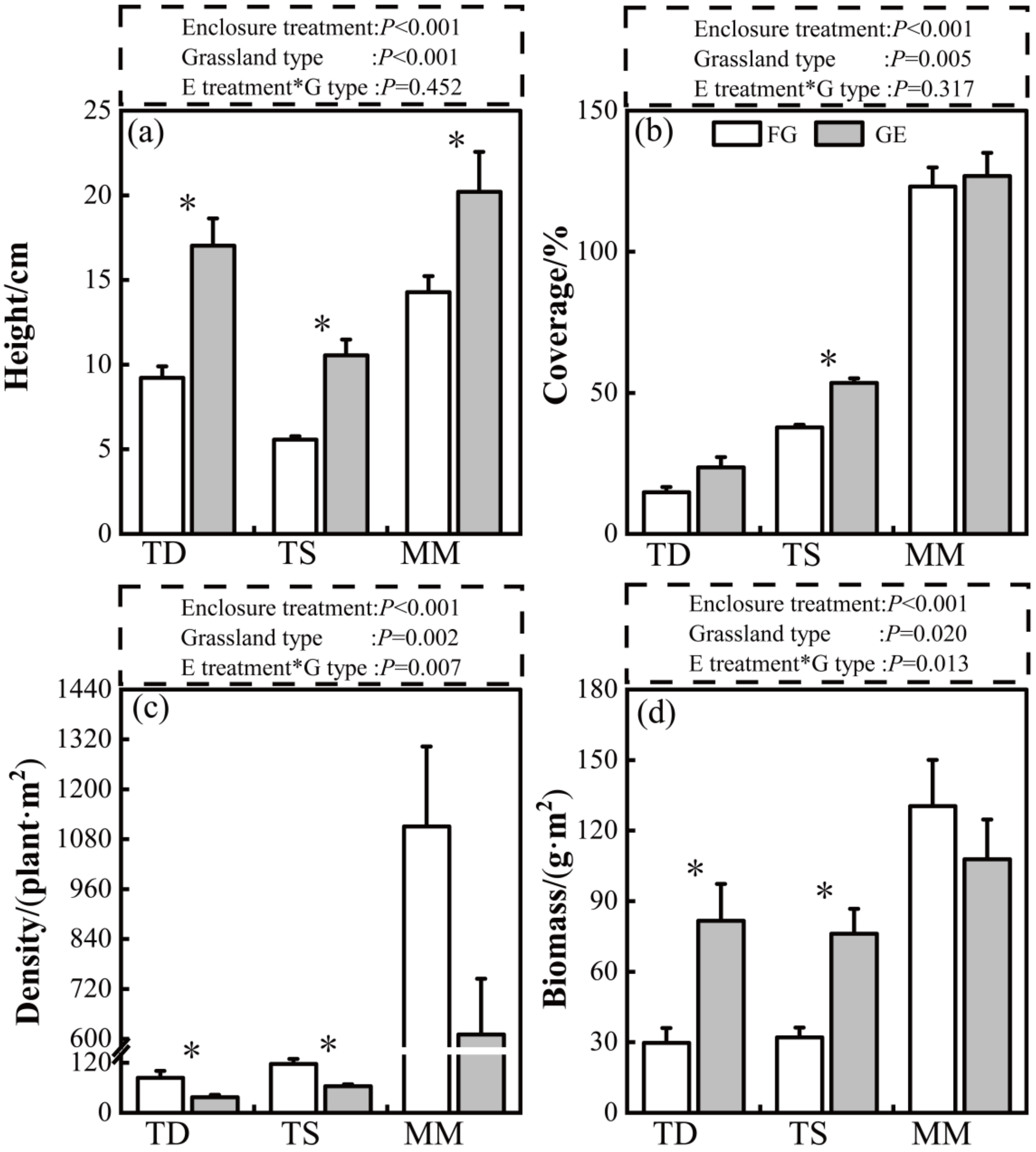

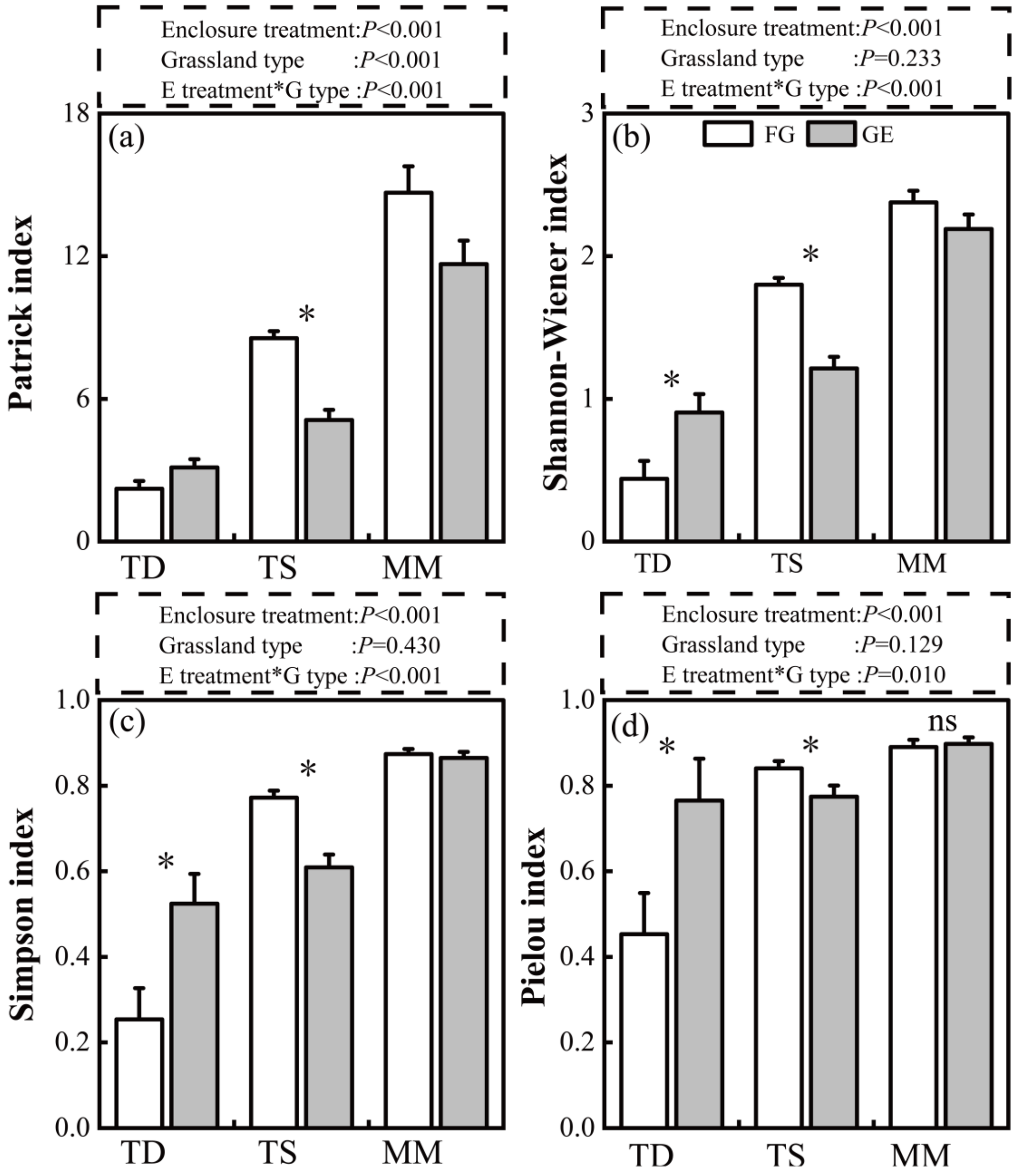

Supplementary Table S1 The differences of fungal major class among three types of grasslands in grazing exclusion sites.

Soil layer(cm)	Grassland type	Enclosure treatment	Archaeorhizomycetes	Dothideomycetes	Eurotiomycetes	Sordariomycetes	Agaricomycetes
0~5	TD	GE	445.67±115.81Aa	9192±1508.79Aa	323.00±226.42Aa	1457.33±1049.34Aa	534.67±180.01Aa
		FG	339.67±102.77Aa	11549.67±2048.73Aa	70.00±9.64Aa	580.67±194.28Aa	396.67±196.39Aa
	TS	GE	125.33±35.22Aa	5104.00±1420.28Aa	620.67±139.74Aa	1303.33±246.82Aa	4486.00±2358.90Aa
		FG	123.33±25.76Aa	4092.33±810.94Ba	603.33±202.01Ba	1268.33±292.92Aa	1497.67±271.30Ba
	MM	GE	8233.67±863.26Ba	623.67±218.84Ba	313.67±33.98Aa	985.33±68.63Aa	1344.33±500.09Aa
		FG	9635.00±1961.45Ba	1077.00±626.70Ba	289.00±35.92ABa	899.00±247.36Aa	818.33±393.93ABa
5~10	TD	GE	222.67±101.33Aa	8552.33±1640.27Aa	457.00±287.83Aa	1539.67±793.90Aa	342.00±49.17Aa
		FG	930.33±303.76Aa	6885.00±1229.19Aa	1103.33±712.63Aa	2339.33±460.78Aa	1410.67±649.75Aa
	TS	GE	90.67±32.54Aa	3140.00±425.58Ba	500.67±103.86Aa	1274.67±586.36Aa	7298.33±3601.22Aa
		FG	165.00±39.17Aa	2832.67±237.53Ba	322.00±50.56Aa	1789.00±966.00Aa	5022.67±3291.68Aa
	MM	GE	7739.33±934.24Ba	472.33±203.93Ba	315.67±146.63Aa	1459.00±520.03Aa	1518.33±765.12Aa
		FG	6824.00±1080.36Ba	538.33±81.28Ba	157.00±71.84Aa	1669.67±547.23Aa	1264.33±323.84Aa

Supplementary Table S2 The differences of soil fungal co-occurrence networks top ological index-es among three types of grasslands in grazing exclusion sites.

Grassland type	Enclosure treatment	Number of nodes	Number of edges	Linkage density
TD	GE	11	9	0.82
	FG	18	15	0.83
TS	GE	28	21	0.75
	FG	24	27	1.13
MM	GE	18	18	1.00
	FG	12	9	0.75
